# From metacognitive beliefs to strategy selection: does fake performance feedback influence cognitive offloading?

**DOI:** 10.1007/s00426-020-01435-9

**Published:** 2020-10-26

**Authors:** Sandra Grinschgl, Hauke S. Meyerhoff, Stephan Schwan, Frank Papenmeier

**Affiliations:** 1 Department of Psychology, University of Tübingen, Schleichstr. 4, 72076 Tübingen, Germany; 2 Leibniz-Institut Für Wissensmedien, Schleichstr. 6, 72076 Tübingen, Germany

## Abstract

The ubiquitous availability of technological aids requires individuals to constantly decide between either externalizing cognitive processes into these aids (i.e. cognitive offloading) or relying on their own internal cognitive resources. With the present research, we investigated the influence of metacognitive beliefs on individuals’ offloading behavior in an experimental setup (*N* = 159). We manipulated participants’ metacognitive beliefs about their memory abilities by providing fake performance feedback: below-average feedback, above-average feedback, or no feedback (control-group). We then measured offloading behavior, using a pattern copying task in which participants copied a color pattern from a model window into a workspace window. While solving this task, participants could rely either more on an internal memory strategy or more on an offloading strategy. Fake performance feedback affected the participants’ metacognitive evaluations about their memory abilities (below-group < control-group < above-group). Although fake performance feedback did not affect actual offloading behavior, the participants receiving below-average performance feedback reported that they had relied more on an offloading strategy than those participants receiving above-average performance feedback. Furthermore, the participants in the below-group reported lower general memory abilities than the other groups at the end of the experiment. We conclude that while fake performance feedback strongly influenced metacognitive beliefs, this did not transfer into a change of strategy selection, thus not influencing offloading behavior. We propose to consider not only metacognitive beliefs but also metacognitive experiences as potential determinants of cognitive offloading.

## Introduction

Today, with regard to the pervasive availability of technological aids such as smartphones or tablets, individuals can constantly decide between either externalizing cognitive processes into these aids by, for example, offloading a shopping list onto one’s smartphone or relying on their own internal cognitive processing by memorizing the shopping list instead. Technological aids serve as a digital expansion of the individual mind (Clark & Chalmers, 1998) and individuals perceive their external memories as part of themselves (Finley, Naaz, & Goh, 2018). The determinants of utilizing either internal cognitive processes or external cognitive resources have been the focus of recent research (e.g., Gilbert, 2015a; Gray, Sims, Fu, & Schoelles, 2006; Grinschgl, Meyerhoff, & Papenmeier, 2020; Risko & Dunn, 2015; Weis & Wiese, 2018). With the present experiment, we probed whether there is a causal relationship between metacognitive beliefs and offloading behavior by manipulating participants’ metacognitive beliefs about their own working memory performance with fake performance feedback.

The externalization of cognitive processes into technological aids is known as cognitive offloading (Risko & Gilbert, 2016). Cognitive offloading reduces demands on internal cognitive processing and thus minimizes cognitive effort when performing a task. Furthermore, due to cognitive offloading, individuals can store and handle more information simultaneously than within the restrictions of their internal memory capacity. In other words, cognitive offloading allows for overcoming capacity limitations of internal cognitive processing such as in working memory (Risko & Gilbert, 2016). With regard to working memory, cognitive offloading avoids the internal encoding or actively holding of information that is present in the immediate environment (Wilson, 2002). Instead, individuals can rely on the environment, for example, using a technological aid to externally store and/or manipulate information and only access the information when needed (Wilson, 2002).

Over the last years, research has identified multiple determinants for offloading behavior (see Risko & Gilbert, 2016, for a review), such as the characteristics of the technological aid and the task at hand. For example, the likelihood of offloading cognitive processes onto a tablet device depends on the responsivity of the device and the smoothness of the control type (Grinschgl et al., 2020). Current research suggests that cognitive offloading is based on cost–benefit considerations (e.g., Gray et al., 2006). When cognitive offloading is associated with low temporal and/or physical costs while interacting with tools, offloading behavior is more pronounced than with high associated costs (e.g., Cary & Carlson, 2001; Gray et al., 2006; Grinschgl et al., 2020). Regarding the task at hand, the information that needs to be processed also influences offloading behavior. For instance, increases in complexity (Schönpflug, 1986), difficulty (Hu, Luo, & Fleming, 2019), or amount of information (Gilbert, 2015a; Morrison & Richmond, 2020; Risko & Dunn, 2015) results in an increased offloading behavior.

Recently, researchers interested in cognitive offloading started considering determinants of cognitive offloading related to the user of technological aids, such as users’ memory capacity or metacognitive beliefs about their own internal abilities. Individuals offloading behavior is more pronounced, the lower their own internal performance is (Gilbert, 2015b; Risko & Dunn, 2015; but see Morrison & Richmond, 2020, for conflicting results). Importantly, however, prior research suggests that not only objective memory abilities but also metacognitive beliefs about one’s internal memory abilities and one’s environment might affect offloading behavior (Arango-Muñoz, 2013). In their review article, Risko and Gilbert (2016) proposed a metacognitive model of cognitive offloading. This model states that the decision between internal and external strategies is guided by metacognitive beliefs about one’s environment—such as the properties of technological aids—and one’s internal memory abilities. Regarding the former, that is, the metacognitive beliefs about one’s environment, studies have shown that individuals adapt their offloading behavior according to their beliefs about the benefits of an offloading strategy (Dunn & Risko, 2015) or the reliability of a technological aid (Weis & Wiese, 2018). If individuals expected an offloading strategy to be inefficient for reaching their goal (Dunn & Risko, 2015) or a technological aid to be unreliable (Weis & Wiese, 2018), they offloaded less and relied more on their own internal resources. Regarding the latter—metacognitive beliefs about one’s internal abilities—Gilbert (2015b) observed in a prospective memory task that the subjective confidence in one’s memory performance predicted offloading behavior, regardless of objective accuracy. Lower confidence in one’s memory performance (i.e. less positive metacognitive evaluations about one’s memory) was associated with a more extensive use of external reminders, thus more cognitive offloading (Boldt & Gilbert, 2019; Gilbert, 2015b; similar results were obtained by Hu, et al., 2019; Risko & Dunn, 2015). Therefore, individuals might use cognitive offloading as a compensatory strategy if they believe that their internal memory abilities are poor. In a recent experimental study using the same prospective memory task, Gilbert et al. (2020) manipulated the difficulty of practice trials as well as the valence of provided feedback on each trial (positive vs. negative). After performing the practice trials, the participants provided metacognitive performance estimations and then performed the task with the possibility of offloading memory demands. The participants rated their own memory performance to be more accurate when they received positively framed feedback or easier practice trials than when they received negatively framed feedback or more difficult practice trials. This shift in metacognitive evaluations was accompanied by a matching shift in offloading behavior. When a manipulation resulted in less confidence in one’s memory abilities, this led to more cognitive offloading. However, all participants showed a bias towards using cognitive offloading extensively, thus metacognitions cannot fully explain offloading behavior in this study (Gilbert et al., 2020). While these findings are a first indication of the connection between metacognitions and cognitive offloading beyond correlational approaches, further investigations are needed to explain their causal relationship as well as the involved processes.

In the present study, we set out to investigate the causal relationship between metacognitive beliefs and offloading behavior by manipulating metacognitive beliefs with fake performance feedback. Performance feedback can influence motivation (Venables & Fairclough, 2009), effort spent on a task (Raaijmakers, Baar, Schaap, Paas, & Van Gog, 2017) as well as goals (Fishbach, Eyal, & Finkenstein, 2010; Ilies & Judge, 2005), even if the feedback is manipulated and therefore false (Ilies & Judge, 2005). With regard to perceptual learning, fake performance feedback has an even higher impact than genuine feedback (Shibata, Yamagishi, Ishii, & Kawato, 2009). Additionally, positive and negative performance feedback can influence beliefs about one’s self-efficacy (Nease, Mudgett, & Quiñones, 1999). Individuals often evaluate their own performance in comparison to other individuals, such as those in their peer group (Ilies & Judge, 2005; MacFarland & Miller, 1994). Thus, performance feedback including a social comparison (e.g., “you performed worse/better than your peers”) might have a particularly strong effect on metacognitive beliefs. In addition, participants might be less able to judge their own performance in relation to their peers compared to directly estimating their own abilities (without any social comparison). Thus, they might be more vulnerable to fake performance feedback with rather than without social comparisons. For these reasons, we provided the participants of our study with fake performance feedback indicating a below-average or above-average performance compared to their peers (i.e. other students), and we measured participants’ metacognitive evaluations with subjective performance ratings similar to the feedback.

We predicted that fake performance feedback should influence participants’ metacognitive beliefs about their own working memory performance. We further hypothesized that the manipulated metacognitive beliefs should transfer into the control of offloading behavior in a working memory task. Therefore, we expected that the participants receiving below-average performance feedback rely more on cognitive offloading while performing a working memory task than those participants receiving above-average performance feedback, with the control group (i.e. no feedback) in between the two. We expected this effect to be due to metacognitive beliefs about the reliability of the internal working memory resources. Whereas the participants receiving below-average feedback should expect their memory to be poor, thus relying more on offloading, those participants receiving above-average feedback should expect their memory to be good, thus relying more on internal processing.

## Method

We preregistered the research questions, independent and dependent variables, sample size, exclusion criteria, and the analysis plan of this experiment at the Open Science Framework prior to data collection (https://osf.io/9hpz5).

### Participants

We collected valid datasets of 159 participants (113 female, 46 male; age 18–32 years, *M*_age_ = 23.16, SD_age_ = 2.81). According to our preregistered exclusion criteria, we excluded and replaced data of participants when data went missing (3) and when there were errors in data collection (2), participants exceeding threshold values of ± 3 SD for the dependent variables in the Pattern Copy Task (4), participants not performing the Feature Switch Detection Task correctly (i.e. always pressing the same button or performing at chance level; 9) and participants who indicated that they had received other feedback than they actually had at the end of the experiment (i.e. an attention check; 5). We also excluded and replaced one participant who reported difficulties when performing the experiment due to visual impairments. Further, we excluded and replaced 13 participants^1^ due to an error in data collection, resulting in missing responses for the paper-and-pencil multifactorial memory questionnaire. The sample size was preregistered and intended to achieve a statistical power of (1 − *β*) = 0.80 with medium effect sizes of *f* = 0.25. The participants were university students and recruited at the University of Tübingen. All participants provided informed consent and received course credits or a financial compensation for their participation. The study was approved by the local ethics committee of the Leibniz-Institut für Wissensmedien.

### Apparatus

All computer tasks were performed on 12.3” Microsoft Surface Pro Tablets (2736 × 1824 pixels) lying flat on the table at a viewing distance of approximately 36 cm. The tablets were controlled by their touch function, and all computer tasks were performed with PsychoPy scripts (Peirce, 2007).

### Procedure and computer tasks

#### General procedure

At the beginning of the experiment, we instructed the participants that they will perform multiple different working memory tests and that they might receive feedback about their actual task performance. Thus, the participants were naïve to our manipulations; that is, they neither knew that the performance feedback was actually fake, nor did they know that the fourth test was designed to measure offloading behavior. The participants first completed three successive working memory tasks (Feature Switch Detection Task, Adapted Corsi Blocks Task, Adapted Visual Patterns Test). Each task started with the participants reading the corresponding instructions and then filling out a pre-rating about their expected upcoming performance. After each task, fake performance feedback was presented for the below-average group and above-average group (see below for details on the feedback). With these three tasks, we aimed to achieve high credibility of the fake performance feedback. As the fourth test, the participants performed our main task—the Pattern Copy Task—measuring spontaneous offloading behavior. The participants were instructed as if this task would just be another common working memory test so that they would transfer the previous fake performance feedback and the associated metacognitive beliefs onto the Pattern Copy Task. After reading the instructions for the Pattern Copy Task, the participants rated their expected upcoming performance. After performing the task, they additionally rated their achieved performance in a post-rating. In addition, they indicated the strategy they used during this task in a follow-up questionnaire. Finally, the participants answered the multifactorial memory questionnaire (MMQ; Troyer & Rich, 2002). All participants were debriefed at the end of the experiment.

#### Feature Switch Detection Task

**Fig. 1:**
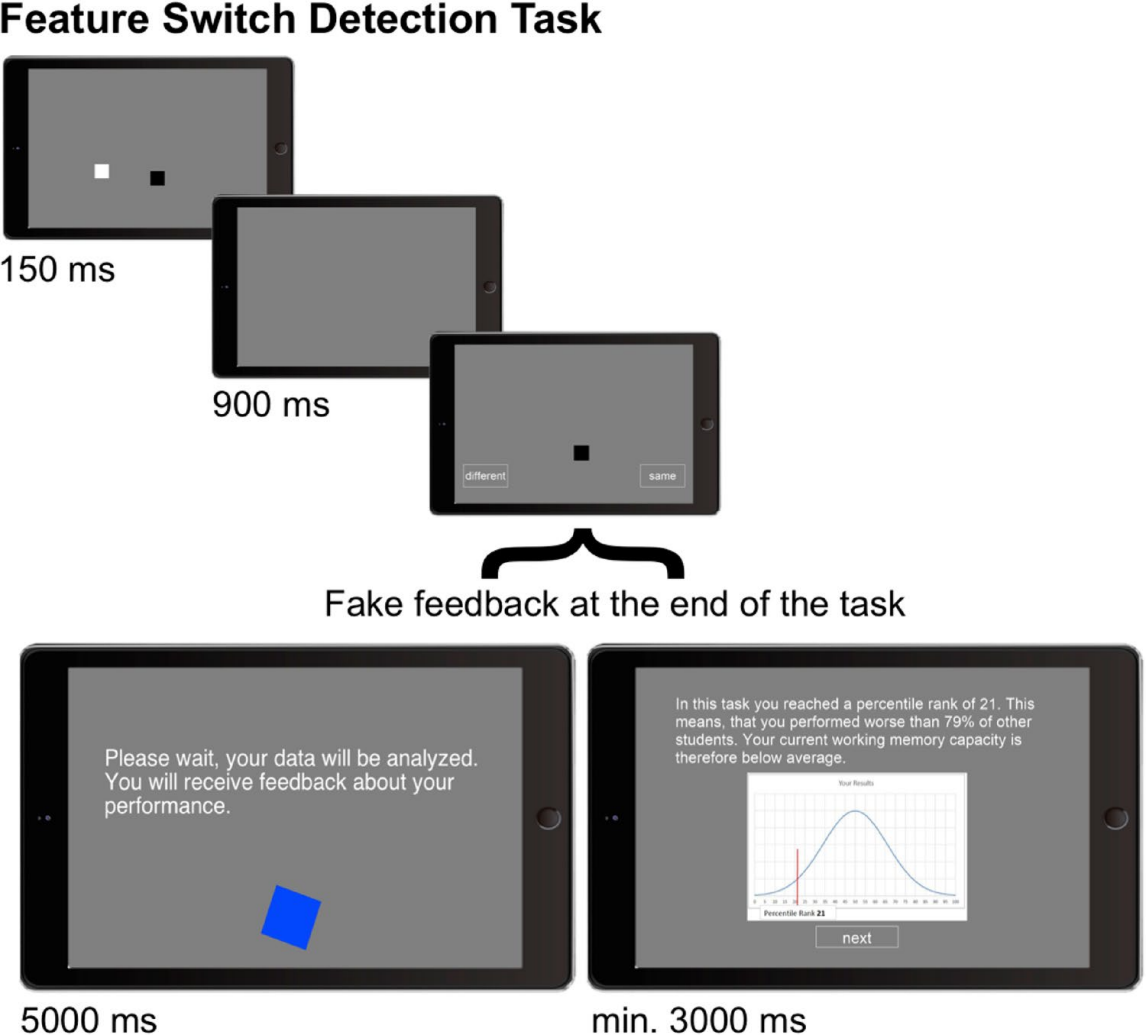
Illustration of the Feature Switch Detection Task, measuring working memory performance. In the Feature Switch Detection Task, the participants had to detect a change in the color of the probed object in a single-probe display. In this example, the color of the probed object did not change; thus, the correct answer is “same” color. After multiple trials with an increasing set size, the participants received fake performance feedback (here illustrated for the below-average group; the tablet frame was designed by Freepik)

We used the Feature Switch Detection Task to measure the participants’ actual visual working memory performance (see Meyerhoff & Gehrer, 2017; Wheeler & Traismen, 2002, for similar versions) and additionally provide our participants with their first fake performance feedback. In this task, the participants had to memorize a display with colored boxes that was presented for 150 ms (presentation display, see Fig. 1). After a short blank period (900 ms), they then observed another display with only one colored box as the probed object (single-probe display) and had to decide whether the color of this probed object was identical to its previous color in the presentation display or whether there was a change in this feature. The single-probe display was presented until a response was given. The participants gave their response by pressing the corresponding button on the touch display (the color of probed object in the single-probe display was the same or was different compared to the presentation display). The task started with eight practice trials including the presentation display of two colored boxes. After the practice trials, the participants performed three blocks of 40 trials with an increasing set size: Block 1 included four colored boxes; Block 2 included six colored boxes, and Block 3 included eight colored boxes in the presentation display. The colored boxes could have had one of the following colors: red, yellow, green, blue, white, brown, black, magenta and were presented on a gray background. Colors were never repeated within a display, and the colored boxes had a size of 2 × 2° of visual angle. In the single-probe display, the probed object either had the same color as in the presentation display (50% of the trials) or it took the color of another object from the presentation display (50% of the trials). The trials were presented in a randomized order within each block. As an index for visual working memory performance, we calculated the proportion of correct responses across all test trials (120 trials in total) per participant.

#### Fake performance feedback

Directly following the task, the participants who received fake performance feedback (below-average group and above-average group) had to wait for 5000 ms in which the computer program (PsychoPy) was pretending to analyze their performance (see Fig. 1). Then, fake performance feedback was presented for at least 3000 ms and until the participants pressed a continue button to exit the feedback. The participants in the control condition did not receive any feedback but instead saw a blue circulating rectangle and the statement “Please wait a moment” (identical to the waiting screen in the other two conditions with exception of the exact statement) on the screen for 8000 ms. They then continued the experiment.

The written feedback gave a fake percentile rank that indicated the performance in the task just performed compared to other students (i.e. their peers) and additionally the meaning of this rank in terms of one’s working memory capacity. More specifically, the feedback after the Feature Switch Detection Task stated the following for the below-average group (in German, here translated into English for illustration):

“In this task, you reached a percentile rank of 21. This means that you performed worse than 79% of other students. Your current working memory capacity is therefore below average.”

For the above-average group, the following fake performance feedback was given subsequently to the Feature Switch Detection Task:

“In this task you reached a percentile rank of 79. This means that you performed better than 79% of other students. Your current working memory capacity is therefore above average.”

In addition, the percentile rank was also visually displayed in a normal distribution. The fake performance feedback for the below-average and above-average groups was consistent across the three tasks for which fake performance feedback was provided. Only the reported values slightly varied. Following the Adapted Corsi Blocks Task, the fake performance feedback for the below-average group was a percentile rank of 23 and for the above-average group a percentile rank of 77. Following the Adapted Visual Patterns Test, the fake performance feedback for the below-average group was a percentile rank of 20, whereas for the above-average group, it was a percentile rank of 80.

#### Adapted Corsi Blocks Task

We presented an adapted Corsi Blocks Task (for original version see Della Sala, Gray, Baddeley, Allamano, & Wilson, 1999) to provide further fake performance feedback. In this task, the participants were presented with a 5 × 5 grid of empty squares (2.52 × 2.52°) on a white display. In a presentation phase, single squares of this grid turned yellow in a specific order (one by one, in a 700 ms rhythm). After the presentation phase and a short blank phase (500 ms), the participants again observed a sequence of single squares turning yellow. They then had to decide whether the second sequence was identical to the first one or not. On 50% of the trials, the sequence was identical; on the other 50% of trials, the sequence was different (one single yellow square was presented in a different position on the grid). The participants performed 36 trials, with the sequence length increasing from four objects turning yellow (12 trials), to six objects turning yellow (12 trials), and finally eight objects turning yellow (12 trials). Subsequently, fake performance feedback was presented according to the participant’s feedback group. We did not analyze actual performance within this task as it only served to provide fake performance feedback.

#### Adapted Visual Patterns Test

This task was also modified from its original version (Della Sala et al., 1999) to provide the participants with fake performance feedback. In this task, the participants had to detect a change between two displays. The displays included a 5 × 5 grid of empty squares (2.52 × 2.52°). Some of these squares were filled with colors in a presentation phase (250 ms). After a short blank phase (1000 ms), the participants observed a second display that was either identical (50% of trials) or the position of one colored square changed (50% of trials; in a randomized order). Thus, the participants had to decide whether the displays were identical or not. In a total of 36 trials, the set size increased, starting with 12 trials with six colored squares each, followed by 12 trials with eight colored squares each, and 12 trials with 10 colored squares each. At the end of this task, the participants received the third and thus last fake performance feedback according to their feedback group. We again did not analyze actual performance within this task.

#### Pattern Copy Task

**Fig. 2:**
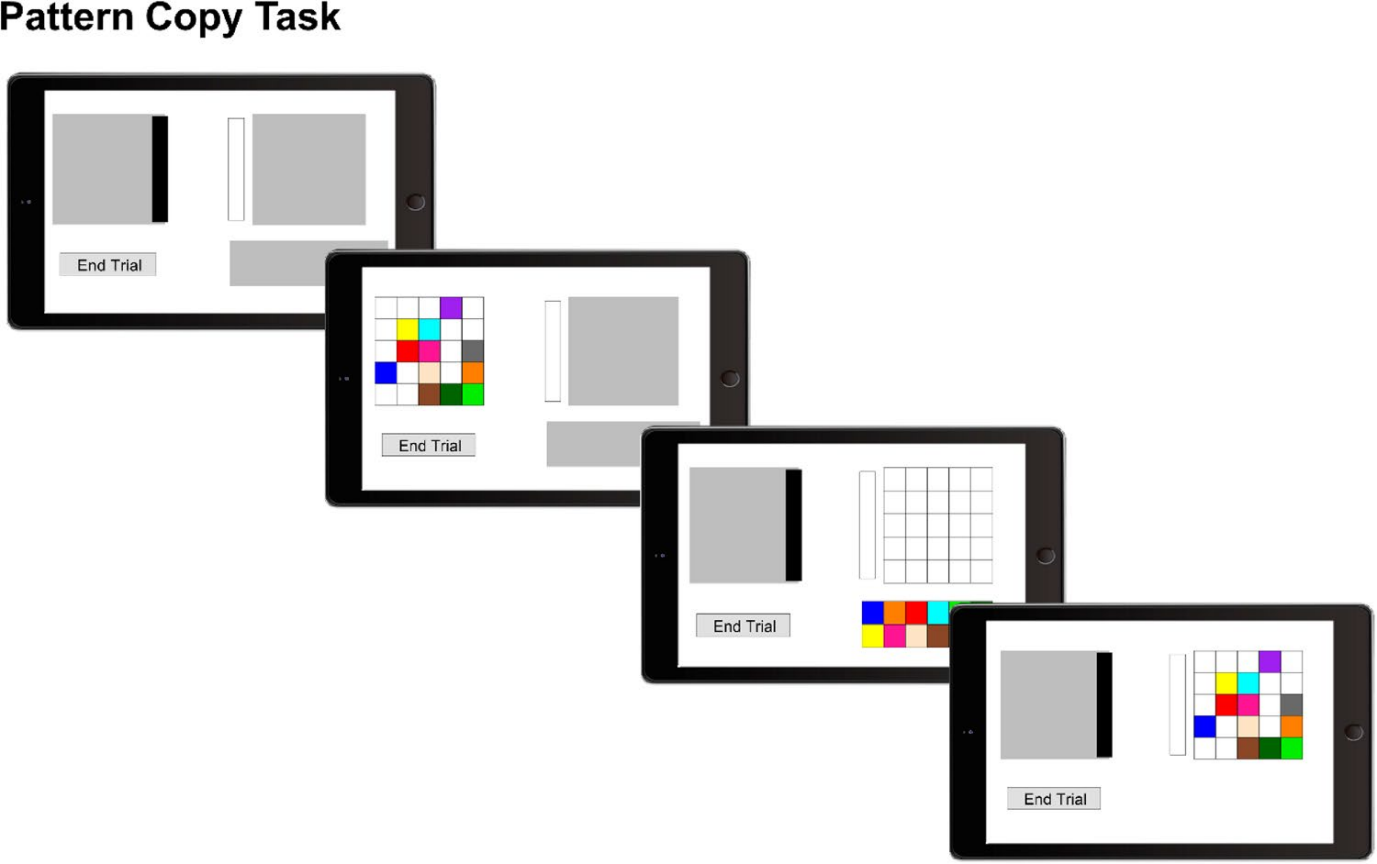
Illustration of the Pattern Copy Task measuring offloading behavior. The participants had to copy a color pattern from a model window (left side) to a workspace window (right side) by dragging colored boxes from an additional resource window (lower right side). The windows were never visible at the same time, but the participants could switch between them as often as they wanted. They could proceed to the next trial once they had correctly rebuilt the pattern. (The tablet frame was designed by Freepik)

The Pattern Copy Task is a working memory task that was designed to measure spontaneous offloading behavior (Ballard, Hayhoe, Li, & Whitehead, 1992; Ballard, Hayhoe, & Pelz, 1995; Gray et al., 2006). The participants were told that they will perform another working memory test that measures their visual working memory capacity (i.e. they did not know about our focus on cognitive offloading). Within this task, the participants had to copy a color pattern from a model window into an empty workspace window (see Fig. 2). The model window comprised a 5 × 5 grid of empty squares (2.52 × 2.52° each). Twelve of these squares were randomly filled with distinct colors (blue, orange, red, cyan, green, dark green, yellow, bisque, sienna, purple, pink, gray; no color was repeated). Thus, the model window presented a color pattern on the left side of the screen that the participants had to reproduce in the workspace window on the right side of the screen. The workspace window presented the same 5 × 5 grid of empty squares, and additionally beneath this workspace window, a resource window was displayed. The resource window contained all the colored boxes to be dragged and dropped into the workspace window. Importantly, all windows were covered by gray masks and only either the model window on the left side of the screen or the workspace and resource window on the right side of the screen could be opened. The model window opened by moving a slider to the left, and the workspace as well as resource window opened by clicking onto a bar next to it. The participants could switch between the windows as often as they wanted. After correctly rebuilding the color pattern in the workspace window, the participants could proceed to the next trial by clicking an “End Trial”-button. If the pattern was not rebuilt correctly, they were requested to keep editing it.^2^ The participants performed 20 trials of this task, preceded by five practice trials. The trial order and color patterns allocated to the trials was randomized to the extent that one participant of each feedback group (below-average, above-average and control group) received the exactly same trial order and color patterns to eliminate potential effects of different stimuli. We measured the offloading of working memory processes with three variables: the number of openings of the model window, the number of correctly copied items after the very first opening of the model window, and the duration of the very first opening of the model window. A higher number of openings indicated more cognitive offloading, whereas more initially correctly copied items and a higher initial encoding duration indicated less cognitive offloading and more memorized information. This task measured spontaneous offloading behavior; thus, the participants were not informed about different strategies (i.e. relying more on cognitive offloading or one’s internal memory) that they might use to solve this task. Instead, the participants had to decide spontaneously which strategy to apply (see also Ballard et al., 1995). This spontaneous offloading behavior resembles the cognitive offloading as performed during daily real-life situations in which individuals usually are also not instructed about specific strategies.

### Paper and pencil tasks

In addition to the computer tasks, we also asked the participants to answer some supplementary measures on paper.

#### Subjective performance ratings

Before performing any computer task, we asked the participants to rate their upcoming performance in comparison to other students. Therefore, they read the instruction of the corresponding task first and then indicated their performance on a 0–100 percentile rank scale. In total, there were four of these pre-ratings (before the Feature Switch Detection Task, the Adapted Corsi Blocks Task, the Adapted Visual Patterns Test and the Pattern Copy Task). For the final Pattern Copy Task, we additionally collected one post-rating. After the completion of this task, the participants rated their actual performance within the Pattern Copy Task on the same scale from percentile 0 to 100.

#### Offloading-strategies

After performing the Pattern Copy Task and filling out the post-rating, the participants were asked about the strategies they used while performing this task. Therefore, we presented the following question and answers to the participants: “What strategy did you use to complete the last task?” with the response options: “I tried to memorize a lot at once instead of having to take a look more often.” or “I tried to take a look more often instead of memorizing a lot at once.” (presented in German; here translated into English for illustration). Thus, the participants could choose between a strategy that implies a more memory intense-strategy (i.e. memorizing more information and looking up the required information less often, “internal strategy”) or more cognitive offloading (i.e. looking up the required information more often and memorizing less information, “offloading strategy”).

#### Multifactorial Memory Questionnaire

At the end of the entire experiment, the participants filled out the MMQ (Troyer & Rich, 2002; adapted and translated by us). This adapted version included statements about meta-memory contentment (18 statements) and omitted other parts of the original version. The statements that we included dealt with the satisfaction with and confidence in someone’s memory abilities, such as “I am generally pleased with my memory ability”. The participants rated how strongly they agreed with these statements on a 5-point Likert scale (from “strongly agree” to “strongly disagree”). We aimed to measure participants’ subjective beliefs about their general memory abilities. Thus, we averaged all 18 ratings to receive an index of subjective beliefs about their general memory abilities (higher values indicate more positive beliefs). Our adapted and translated questionnaire provided a high internal consistency with a Cronbach’s alpha of 0.93.

### Design

Our experiment followed a between-subjects design with three feedback groups (below-average vs. above-average vs. control). In the below-average group, the participants received fake performance feedback indicating below-average working memory capacity. In the above-average group, the participants received fake performance feedback indicating above-average working memory capacity. The control group did not receive any feedback at all.

## Results

### Subjective performance ratings

**Fig. 3:**
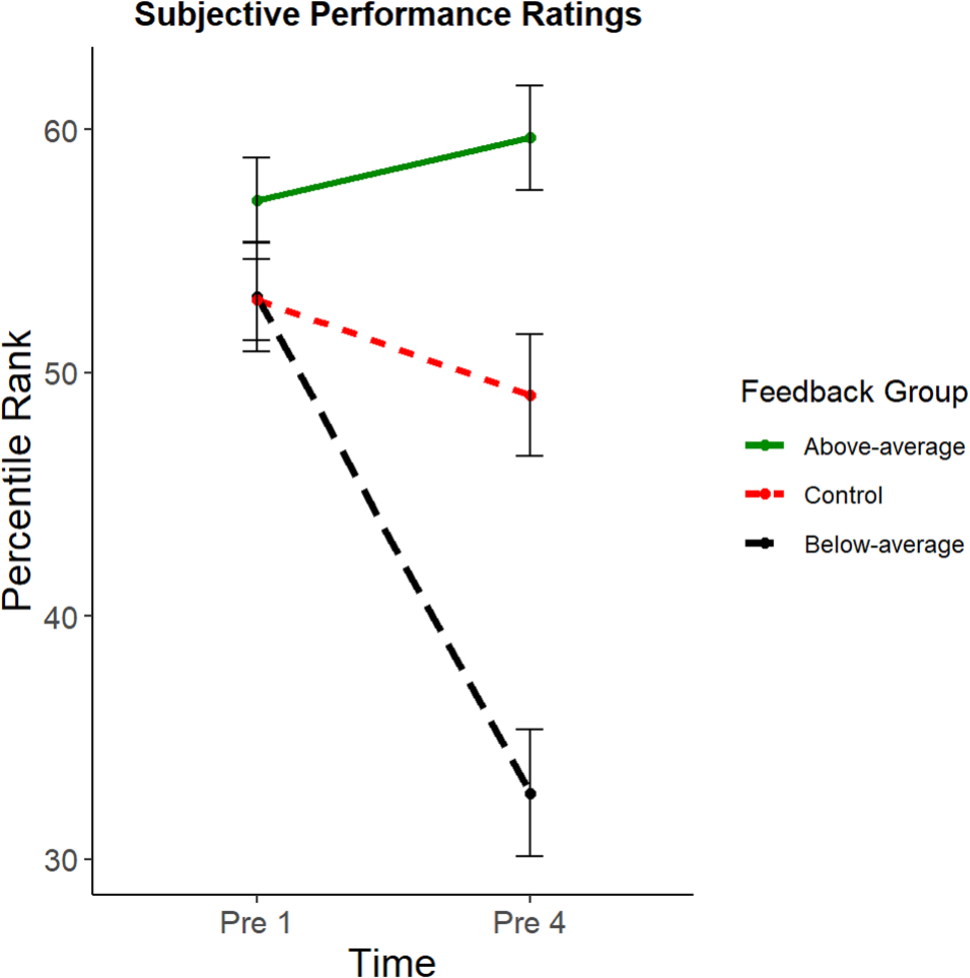
Subjective performance ratings prior to receiving fake performance feedback (Pre 1) and after receiving multiple fake performance feedback (Pre 4; with standard errors of the mean as error bars). We observed no group differences at the first pre-rating before receiving fake performance feedback and significant differences between all feedback groups at the fourth pre-rating after receiving fake performance feedback and before performing the Pattern Copy Task

To investigate whether the participants changed their metacognitive evaluations according to the provided fake performance feedback, we performed a preregistered mixed 2 × 3 ANOVA with the within factor “time of pre-rating” (first pre-rating before receiving any feedback vs. fourth pre-rating after receiving feedback for three times) and the between factor “feedback group” (below-average vs. above-average vs. control). We were especially interested in the fourth pre-rating as it was provided immediately before the main task (Pattern Copy Task) measuring cognitive offloading. We observed a main effect of the factor “feedback group”, *F*(2, 156) = 19.74, *p* < 0.001, *η*^2^ = 0.12, as well as a main effect of the factor “time of pre-rating”, *F*(1, 156) = 22.67, *p* < 0.001, *η*^2^ = 0.04. Most importantly, we also found a significant interaction between these factors, *F*(2, 156) = 20.09, *p* < 0.001, *η*^2^ = 0.07. Post-hoc *t* Tests for independent samples between each feedback group showed that there were no group differences in the first pre-rating, all *t*s(104) <  = 1.68, all *p*s >  = 0.094, all *d*s <  = 0.33, whereas all groups differed from each other in the fourth pre-rating, all |*t*s(104)|> = 3.21, all *p*s <  = 0.001, all |*d*s|> = 0.62 (see Fig. 3). Thus, as expected, at the very first pre-rating before receiving any fake performance feedback, the participants did not differ in their metacognitive evaluations about their upcoming performance, but after receiving fake performance feedback three times, the below-average group indicated the lowest performance, whereas the above-average group indicated the highest performance, with the control group in the middle.^3^

### Cognitive offloading

We performed preregistered one-way ANOVAs for the between factor “feedback group” (below-average vs. above-average vs. control) and each of the three dependent offloading variables of the Pattern Copy Task. The three feedback groups did not differ significantly in the number of times the model window was opened, *F*(2, 156) = 1.04, *p* = 0.354, *η*^2^ = 0.01, the number of initially correctly copied items, *F*(2, 156) < 0.01, *p* = 0.998, *η*^2^ < 0.01, and the initial encoding duration, *F*(2, 156) = 0.97, *p* = 0.383, *η*^2^ = 0.01 (see Table 1). Thus, our fake feedback manipulation did not alter offloading behavior in the Pattern Copy Task. For exploratory purposes, we also analyzed the trial duration within the Pattern Copy Task as an indicator of task performance. The three feedback groups did not differ in the trial duration, *F*(2, 156) = 0.73, *p* = 0.486, *η*^2^ = 0.01 (see Table 1).

**Table 1:** Means and standard deviations of dependent variables in cognitive offloading as well as trial duration in the Pattern Copy Task and working memory performance in the Feature Switch Detection Task

	Below-average group	Control group	Above-average group
	*M* (SD)	*M* (SD)	*M* (SD)
Cognitive offloading			
Openings of the model window	5.39 (1.08)	5.14 (1.02)	5.09 (1.25)
Initially correctly copied items	3.26 (0.61)	3.26 (0.74)	3.27 (0.71)
Initial encoding duration (sec)	7.21 (3.52)	7.17 (4.53)	6.33 (2.83)
Trial duration (sec)	43.45 (8.49)	41.42 (10.10)	41.73 (9.38)
Working memory performance	0.74 (0.07)	0.73 (0.07)	0.73 (0.09)

### Offloading-strategies

Following the Pattern Copy Task, the participants stated which strategy they had preferred during this task. They could either choose a strategy that indicated more cognitive offloading (“offloading strategy”) or a strategy that indicated more internal cognitive processing (“internal strategy”). The participants that indicated both strategies were excluded from this exploratory analysis (remaining participants: *N* = 146, see Table 2). We used a logistic regression and the Anova-function from the car package (Fox & Weisberg, 2019) to analyze the differences in the selected strategies across the three feedback groups. There was a significant main effect of feedback group on the selected strategies, *X*^2^(2) = 10.39, *p* = 0.005, *d* = 0.55, indicating that the fake performance feedback had affected the participant’s choice of which strategy they thought they had used during the Pattern Copy Task. We used reduced logistic regressions, including only two feedback groups each to calculate pairwise comparisons. This comparison revealed that the participants from the below-average condition indicated that they had preferred an offloading strategy over an internal strategy to a larger extent than the participants from the above-average condition, *X*^2^(1) = 10.39, *p* = 0.001, *d* = 0.69. Thus, despite not having observed an objective change in offloading behavior in our Pattern Copy Task, the participants on average reported that they had shifted their strategy in the direction that we had predicted. Further, despite the fact that the numerical frequencies indicate that the control group was right between the below-average condition and above-average condition, those paired comparisons did not reach significance, *X*^2^(1) = 2.90, *p* = 0.088, *d* = 0.35, and *X*^2^(1) = 2.34, *p* = 0.126, *d* = 0.31, respectively.

**Table 2:** Participants per group that indicated using either an “offloading strategy” or an “internal strategy” in the Pattern Copy Task

	Below-average group	Control group	Above-average group
	*N*	*N*	*N*
Offloading strategy	36	29	23
Internal strategy	11	19	28

Additionally, we calculated exploratory point-biserial correlations between strategy selection and actual offloading behavior (see Table 3). Strategy selection and offloading were not significantly correlated in the below-average group. In the above-average group, they were also no significant correlations with the exception of the initial encoding duration. In the control group, however, strategy selection did correlate significantly with cognitive offloading, all |*r*s(46)*|*> = 0.40, all *p*s <  = 0.005. Overall, this analysis indicates that self-reported strategies matched actual performance only in the control group, but not in the groups with experimental manipulations of metacognitive beliefs about one’s own memory performance.

**Table 3:** Point-biserial correlations between reported strategy selection (0 = internal strategy, 1 = offloading strategy) and actual offloading behavior

	Below-average group	Control group	Above-average group
	*r* (45)	*r* (46)	*r* (49)
Openings of the model window	0.07	0.40**	0.01
Initially correctly copied items	− 0.19	− 0.39**	− 0.09
Initial encoding duration (sec)	0.06	− 0.53***	− 0.31*

*p < 0.05, **p < 0.01, ***p < 0.001

### MMQ

**Fig. 4:**
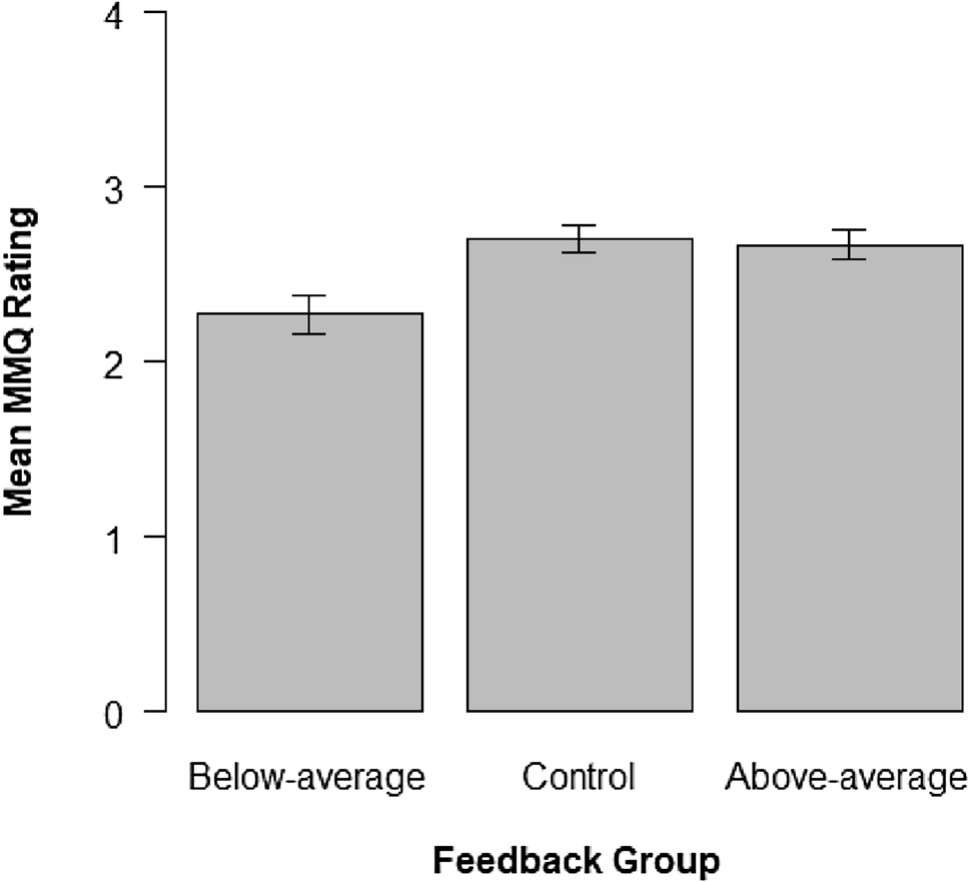
Ratings of beliefs about one’s general memory abilities measured with the MMQ at the end of the experiment (averaged for each group; with standard errors of the mean as error bars). Higher values indicate more positive beliefs about one’s general memory abilities. The below-average group indicated worse general memory abilities than the other two groups

An exploratory one-way ANOVA revealed significant group differences in beliefs about one’s general memory abilities at the end of the experiment, *F*(2, 156) = 7.08, *p* = 0.001, *η*^2^ = 0.08. Additional *t* Tests for independent samples showed that the below-average group rated their general memory abilities lower than the above-average group, *t*(104) = 2.98, *p* = 0.003, *d* = 0.58, and the control group, *t*(104) = 3.19, *p* = 0.002, *d* = 0.62. We observed no significant difference between the above-average group and the control group, *t*(104) = 0.25, *p* = 0.801, *d* = 0.05 (see Fig. 4). Thus, our fake performance feedback manipulation altered the participants’ beliefs about their general memory abilities in the direction of the feedback provided, particularly for the below-average group.

### Working memory capacity

To exclude that any group effects are due to differences in actual working memory abilities, we used the Feature Switch Detection Task to measure visual working memory performance for color-location bindings. A preregistered one-way ANOVA indicated that there were no significant group differences in this working memory performance measure, *F*(2, 156) = 0.07, *p* = 0.929, *η*^2^ < 0.01 (see Table 1), just as one would expect given the randomized assignment of the participants to the experimental conditions.

## Discussion

In the present study, we investigated the causal impact of metacognitive beliefs about one’s working memory on cognitive offloading in a working memory task. Metacognitive beliefs are supposed to influence one’s decision for using specific strategies when performing a task based on metacognitive monitoring and control. Thus, metacognitive beliefs should affect the use of technological aids (and likewise cognitive offloading) or one’s internal working memory resources. To experimentally test the determining role of metacognitive beliefs when offloading working memory processes, we used fake performance feedback. Our fake performance feedback successfully manipulated metacognitive beliefs about one’s working memory. Before receiving any feedback, the three feedback groups did not differ in their pre-rating about their upcoming working memory performance, but after receiving fake performance feedback, they differed accordingly. The participants receiving below-average performance feedback rated their working memory performance the lowest, and those participants receiving above-average performance feedback rated their working memory performance the highest, with the control group that did not receive any feedback in between. Remarkably, the effect of fake performance feedback was so strong that it even spilled over to general beliefs about one’s memory abilities (measured by the MMQ) at the end of the experiment. The participants in the below-average group estimated their general memory abilities lower than the other two groups. Thus, especially below-average performance feedback affected metacognitive beliefs broadly and persistently. Although our manipulation of metacognitive beliefs altered the participants’ subjective working memory ratings, it had clearly no impact on offloading behavior within the Pattern Copy Task. Within this task, participants could either rely more on a technological aid by looking up information more often (i.e. more cognitive offloading) or rely more on their own internal memory by looking up the information less often (i.e. less cognitive offloading). We observed that spontaneous offloading behavior within the Pattern Copy Task was nearly identical across all feedback groups.

Previous research suggests that metacognitions are negatively correlated with offloading behavior (Boldt & Gilbert, 2019; Gilbert, 2015b; Hu, et al., 2019; Risko & Dunn, 2015). For instance, in studies applying a prospective memory paradigm (Gilbert, 2015b; see also Boldt & Gilbert, 2019; Gilbert et al., 2020), the participants had to drag circles with ascending numbers one after another to the bottom of the screen. At the beginning of a trial, the participants were instructed that some special circles (e.g., the circle with the number 3) had to be dragged to another side of the screen (e.g., the left side) when it was their turn. These special circles induced intentions that the participants needed to fulfill later on. After performing practice trials, the participants were asked to rate their upcoming performance (0–100% of special circles dragged to the correct location). The participants then performed several trials of the task without the option to offload, followed by several trials that allowed cognitive offloading. In these latter trials the participants could offload the intentions by placing the special circles close to the correct side of the screen already at the beginning of a trial. More positive evaluations about one’s unaided memory performance were associated with less cognitive offloading (Boldt & Gilbert, 2019; Gilbert, 2015b; see also Hu et al., 2019; Risko & Dunn, 2015, for similar results). While these correlational findings suggest a relationship between metacognitions and offloading behavior, we did not observe a matching impact of metacognitions on cognitive offloading in the present experimental study. To resolve these seemingly conflicting results, we suggest that the differentiation of metacognitions into metacognitive beliefs and metacognitive experiences might explain the diverging results across studies.

In the present study, we manipulated metacognitive beliefs by providing the participants with fake performance feedback on three different working memory tasks but—importantly—before they gained any actual experience in performing the Pattern Copy Task. In contrast, previous research reporting significant effects of metacognitions on offloading behavior collected metacognitive performance estimations after participants performed practice trials of the offloading task (Boldt & Gilbert, 2019; Gilbert, 2015b) or the presentation of the relevant stimuli (Hu, et al., 2019; Risko & Dunn, 2015). Theoretical accounts of metacognitions often differentiate between metacognitive beliefs—referring to general beliefs about one’s person stored in long-term memory (e.g., beliefs about one’s memory abilities)—and metacognitive experiences—referring to task-specific knowledge that is present before, during, or while performing a cognitive task (Efklides, 2008; Flavell, 1979). Metacognitions manipulated in our study were supposed to reflect the former—general beliefs about one’s working memory based on fake performance feedback in other tasks. On the other hand, metacognitions measured in the previous studies (e.g., Gilbert, 2015b) rather reflect the latter—metacognitive experiences—due to the measurement after performing practice trials, for instance. This latter design was also used in a recent study showing that the manipulated valence of feedback on task trials influenced metacognitions and in return offloading behavior (Gilbert et al., 2020). Therefore, we suggest that it might actually be metacognitive experiences (as measured by the previous studies) rather than metacognitive beliefs (as manipulated in our study) that drive offloading behavior.

The suggestion that metacognitive experiences rather than metacognitive beliefs alter offloading behavior fits in well with research showing that actual offloading behavior is determined by the properties of the task at hand and thus probably metacognitive experiences. In like manner, cognitive offloading is known to be driven by external factors such as tool design (Grinschgl et al., 2020) costs when interacting with external tools (e.g., Cary & Carlson, 2001; Gray et al., 2006; Grinschgl et al., 2020), or characteristics of processed information (Gilbert, 2015a; Hu et al., 2019; Morrison & Richmond, 2020; Risko & Dunn, 2015; Schönpflug, 1986). Such external factors are likely to influence metacognitive experiences while performing a task and in turn influence offloading behavior. Within this context, the new finding of our study is that metacognitive beliefs in contrast to metacognitive experiences had no influence on offloading behavior—at least not within the Pattern Copy Task.

Interestingly, we observed an influence of fake performance feedback on subjective judgements regarding the offloading strategy in the Pattern Copy Task. The participants in the below-average group were more likely to report an offloading strategy over an internal strategy than the participants from the above-average group, although their actual offloading behavior was nearly identical. The distinction between metacognitive beliefs and metacognitive experiences also provides a way to resolve the apparent contradiction between perceived and actual strategy use. Whereas metacognitive experiences could be the main determinant of actual offloading behavior in the Pattern Copy Task, participants might rather consider their metacognitive beliefs when giving subjective judgements on their behavior. For instance, negative beliefs about one’s performance might lead participants to judge their behavior as offloading more (although they actually did not offload more) than positive beliefs about one’s performance. Thus, based on metacognitive beliefs, the same actual behavior might be interpreted differently by the participants. This assumption was further supported by exploratory correlations showing that the reported strategy selection did not correlate with the actual offloading behavior in the below-average group as well as in the above-average group across most offloading-variables. Interestingly, however, in the control group we did indeed observe such a correlation; that is, participants that reported to have used an offloading strategy also offloaded more within the Pattern Copy Task. Thus, without a manipulation of metacognitive beliefs with fake performance feedback, participants could correctly judge their own performance.

When relating the findings of our present study to previous research, it is also important to consider the differences between the offloading tasks applied. For instance, in a prospective memory task that has established a correlation between metacognitions and cognitive offloading (Boldt & Gilbert, 2019; Gilbert, 2015b), the participants offloaded future intentions, whereas in the Pattern Copy Task the participants offloaded by looking up relevant information. These two kinds of offloading behavior might be different per se, thus also be guided by different determinants. It is possible that fake performance feedback and in return metacognitive beliefs would indeed drive the offloading of intentions in a prospective memory task, but not offloading behavior in the Pattern Copy Task. We can only speculate about the different processes involved in these offloading paradigms as no study has directly compared them. However, one important difference might be the involved timing when offloading memory processes. Whereas in studies using the prospective memory task (Boldt & Gilbert, 2019; Gilbert, 2015b; Gilbert et al., 2020), the participants offloaded future intentions (i.e. the information is offloaded for remembering it later on), in the Pattern Copy Task the participants offloaded information for instantaneous use (i.e. looking up information more often for the ongoing copy task). Thus, offloading of future intentions might be related to planning before actual task performance, while offloading in the Pattern Copy Task might be related to ongoing processes throughout the task.^4^ Metacognitive beliefs could possibly play a greater role for planning before action (thus affecting the offloading of intentions) rather than for offloading during ongoing task processing. Further research is needed to investigate the different as well as shared processes involved in cognitive offloading across various paradigms.

Another difference between previous studies investigating metacognitions as determinant of cognitive offloading (e.g., Gilbert, 2015b) and the present study is the specific framing of participants’ performance estimations. While in previous studies the participants estimated their own performance based on how accurate they think their own performance is (0–100% accuracy; Boldt & Gilbert, 2019; Gilbert, 2015b; Gilbert et al., 2020; Risko & Dunn, 2015), in our experiment they estimated their performance in comparison to other students via a percentile rank. This latter estimation in our study was in line with the provided fake performance feedback that was designed to have a strong impact due to social comparisons (MacFarland & Miller, 1994). It might be argued that cognitive offloading was guided rather by metacognitive beliefs without any comparison (i.e. the participants might adopt their offloading behavior based in their confidence in their own memory, independent of its relation to other individuals). However, in our study we also measured metacognitive beliefs with the MMQ that did not include any estimations compared to other individuals and—importantly—our manipulation also affected the metacognitive beliefs as measured in this questionnaire following below-average performance feedback. We thus consider it unlikely that the specific framing of the fake performance feedback as well as performance estimations was a key factor in explaining differences in results between our present study and previous research on cognitive offloading.

The participants in the below-average group estimated their general memory abilities lower than the other two feedback groups. Thus, it seems that below-average performance feedback has a particularly strong influence on metacognitive beliefs and self-perception. In a similar vein, Davis and Brock (1975) showed that below-average performance feedback influences the participants’ self-awareness compared to no feedback or above-average feedback, while the latter two conditions did not differ from each other. However, it might not only be below-average feedback per se that strongly influences metacognitive beliefs. Another possibility could be that below-average feedback induces a large deviation from one’s primary beliefs before receiving feedback. For instance, one might think that his or her performance is slightly above average. In this case, receiving above-average feedback suggesting a percentile rank of 79% might be less unexpected and thus have less impact than below-average feedback suggesting a percentile rank of 21%, which might largely deviate from one’s primary beliefs. Nonetheless, our findings suggest that below-average performance feedback is particularly suited to experimentally manipulate the participants’ self-perception – an important insight for future experiments.

## Conclusion

Our study aimed to experimentally test the causal impact of metacognitive beliefs about one’s working memory performance as manipulated by fake performance feedback on the offloading of working memory processes with modern technological tools. While fake performance feedback successfully altered participants’ evaluations regarding their performance on the tasks at hand, as well as their beliefs about their general memory abilities, we did not observe a change in actual offloading behavior. We propose that that this putative discrepancy can be resolved by taking the distinction between metacognitive beliefs and metacognitive experiences into account. Whereas participants’ subjective ratings might be largely influenced by their metacognitive beliefs, actual offloading behavior might largely depend on metacognitive experiences and properties of the task at hand—at least within the Pattern Copy Task. Thus, performing future research investigating the influence of metacognitive beliefs and experiences on both strategy selection before starting a task and while performing a task across different offloading paradigms will help to generate a more complex and broadly applicable metacognitive model of cognitive offloading.

## Availability of data and material

The experiment reported in this article was preregistered. The preregistration, data, and materials have been made available on the Open Science Framework: https://osf.io/zrqxu/

## Data Availability

The scripts used for analyzing our data are also available on the Open Science Framework (https://osf.io/zrqxu/).

## Footnotes

1 Please note that exploratorily adding the complete datasets of those participants to the respective analyses changes neither the reported result patterns nor interpretations.

2 Please note that the participants rarely pressed the “End Trial”-button whenever the pattern was not correctly rebuilt. Across the 20 trials of the Pattern Copy Task, we observed the following means and standard deviations for the number of times the “End Trial”-button was pressed prematurely: below-average group: *M* = 0.29 (SD = 0.36); control group: *M* = 0.19 (SD = 0.15); above-average group: *M* = 0.20 (SD = 0.14). Excluding the trials in which participants pressed the “End Trial”-button prematurely did not change our findings.

3 An exploratory mixed 5 × 3 ANOVA including all four pre-ratings as well as the post-rating after performing the Pattern Copy Task and the three feedback groups revealed the same pattern of results for the dependent variable “subjective performance ratings”, all *F*(2, 156)s >  = 10.29, all *p*s < 0.001, all η^2^s >  = 0.03. There were no group differences at the very first rating (Pre 1), but all groups differed in their performance ratings already after receiving fake performance feedback once (i.e. group differences in Pre 2, Pre 3, Pre 4; all |*t*s(104)|> = 3.21, all *p*s <  = 0.001, all |*d*s|> = 0.62) and even after performing the Pattern Copy Task (i.e. post-rating; all |*t*s(104)|> = 3.34, all *p*s <  = 0.001, all |*d*s|> = 0.65). Participants in the below-average group rated their performance lower than participants in the above-average group with the control group in the middle.

4 We would like to thank an anonymous reviewer for suggesting this idea.
